# Ex vivo multi-electrode analysis reveals spatiotemporal dynamics of ictal behavior at the infiltrated margin of glioma

**DOI:** 10.1016/j.nbd.2019.104676

**Published:** 2019-11-12

**Authors:** Brian J.A. Gill, Xiaoping Wu, Farhan A. Khan, Alexander A. Sosunov, Jyun-you Liou, Athanassios Dovas, Tahra L. Eissa, Matei A. Banu, Lisa M. Bateman, Guy M. McKhann, Peter Canoll, Catherine Schevon

**Affiliations:** a Department of Neurological Surgery, Columbia University Medical Center, New York, NY, USA; b Department of Physiology and Cellular Biophysics, Columbia University, New York, NY, USA; c Department of Pathology and Cell Biology, Columbia University Medical Center, New York, NY, USA; d Department of Applied Mathematics, University of Colorado at Boulder, Boulder, CO, USA; e Department of Neurology, Columbia University Medical Center, New York, NY, USA

**Keywords:** Glioma, Epilepsy, Seizure, Microelectrode array, Zero magnesium

## Abstract

The purpose of this study is to develop a platform in which the cellular and molecular underpinnings of chronic focal neocortical lesional epilepsy can be explored and use it to characterize seizure-like events (SLEs) in an ex vivo model of infiltrating high-grade glioma. Microelectrode arrays were used to study electrophysiologic changes in ex vivo acute brain slices from a PTEN/p53 deleted, PDGF-B driven mouse model of high-grade glioma. Electrode locations were co-registered to the underlying histology to ascertain the influence of the varying histologic landscape on the observed electrophysiologic changes. Peritumoral, infiltrated, and tumor sites were sampled in tumor-bearing slices. Following the addition of zero Mg^2+^ solution, all three histologic regions in tumor-bearing slices showed significantly greater increases in firing rates when compared to the control sites. Tumor-bearing slices demonstrated increased proclivity for SLEs, with 40 events in tumor-bearing slices and 5 events in control slices (*p*-value = .0105). Observed SLEs were characterized by either low voltage fast (LVF) onset patterns or short bursts of repetitive widespread, high amplitude low frequency discharges. Seizure foci comprised areas from all three histologic regions. The onset electrode was found to be at the infiltrated margin in 50% of cases and in the peritumoral region in 36.9% of cases. These findings reveal a landscape of histopathologic and electrophysiologic alterations associated with ictogenesis and spread of tumor-associated seizures.

## 1. Introduction

Tumor associated epilepsy (TAE) is one of the most common and devastating types of lesional epilepsy. TAE occurs in 25–60% of high-grade gliomas (HGGs) (Englot et al., 2016), and considerably higher rates are seen in lower grade glial tumors (Chang et al., 2008). These events contribute significantly to the cognitive deterioration and morbidity associated with the disease (van Breemen et al., 2007). Gross total resection achieves primary oncologic goals, but seizures may persist. Intracranial EEG implantation in cases of TAE has demonstrated that the epileptic zone often encompasses regions > 1.5 cm from the tumor margin, in regions where there may be no distinct MRI abnormalities (Hamer and Hong, 2013; Mittal et al., 2016). This suggests a need for a more comprehensive understanding of the electrophysiologic and histopathologic changes induced by the tumor at the infiltrated margin and in the peritumoral cortex. These changes are patchy, ill-defined, and variable, thus making it difficult to understand the relationship between the two (Awad et al., 1991; Barajas Jr. et al., 2012; Eidel et al., 2017; Gill et al., 2014; Mittal et al., 2016; Tran et al., 1997). This challenge can be addressed with dense microelectrode arrays (MEAs), combined with analytical techniques for elucidating seizure localization and propagation, and co-registration to the surrounding histology. MEAs have been used to map neuronal firing patterns during ictal events in vivo(Liou et al., 2018; Schevon et al., 2008; Schevon et al., 2012; Truccolo et al., 2011) and in vitro(Hilgen et al., 2017; Hsiao et al., 2015; Serafini et al., 2016; Shi et al., 2014), offering spatiotemporal resolution and a range of neural activity recording otherwise not achieved with simultaneous patch clamp recordings or voltage sensitive dyes.

A fundamental barrier to characterizing focal seizure dynamics is the lack of consensus regarding the role of specific electrophysiological behaviors. A consistent feature of ictal events, reported in computational(Beverlin II et al., 2012), slice(Trevelyan et al., 2006; Trevelyan et al., 2007) and in vivo models(Liou et al., 2018), and described in humans(Schevon et al., 2012; Smith et al., 2016) is a slowly expanding, focal seizure territory with a consistent pathognomonic electrographic signature: a transient band of tonic firing followed by repetitive clonic bursting synchronized across the territory. The tonic band of firing termed the ictal wavefront, progresses across the cortical territory at speeds ranging from 0.1 to 1 mms^−1^ as measured in a small number of slice studies(Trevelyan et al., 2007; Wong and Prince, 1990) and in human microelectrode array recordings (Schevon et al., 2012). In the territory ahead of the wavefront and in the wavefront itself, there is a notable mismatch between multiunit firing and the local field potentials that contribute largely to clinical EEG recordings (Schevon et al., 2012). These distortions make it difficult to study ictal propagation using wideband electrophysiologic data. With rare exceptions (Hoffman et al., 1994; Jacobs et al., 1999), these ex vivo efforts to investigate ictogenesis and propagation are usually focused on acute pharmacologically induced models(Chan et al., 2019; Curia et al., 2008; Levesque and Avoli, 2013; Losi et al., 2016) as opposed to chronic focal lesional models such as TAE. Here we report findings from MEA recordings in ex vivo acute brain slices from a murine glioma model, relating the spatial seizure structure to the underlying histological milieu.

## 2. Methods

### 2.1. Mouse tumor model and slice preparation

**Fig. 1.**
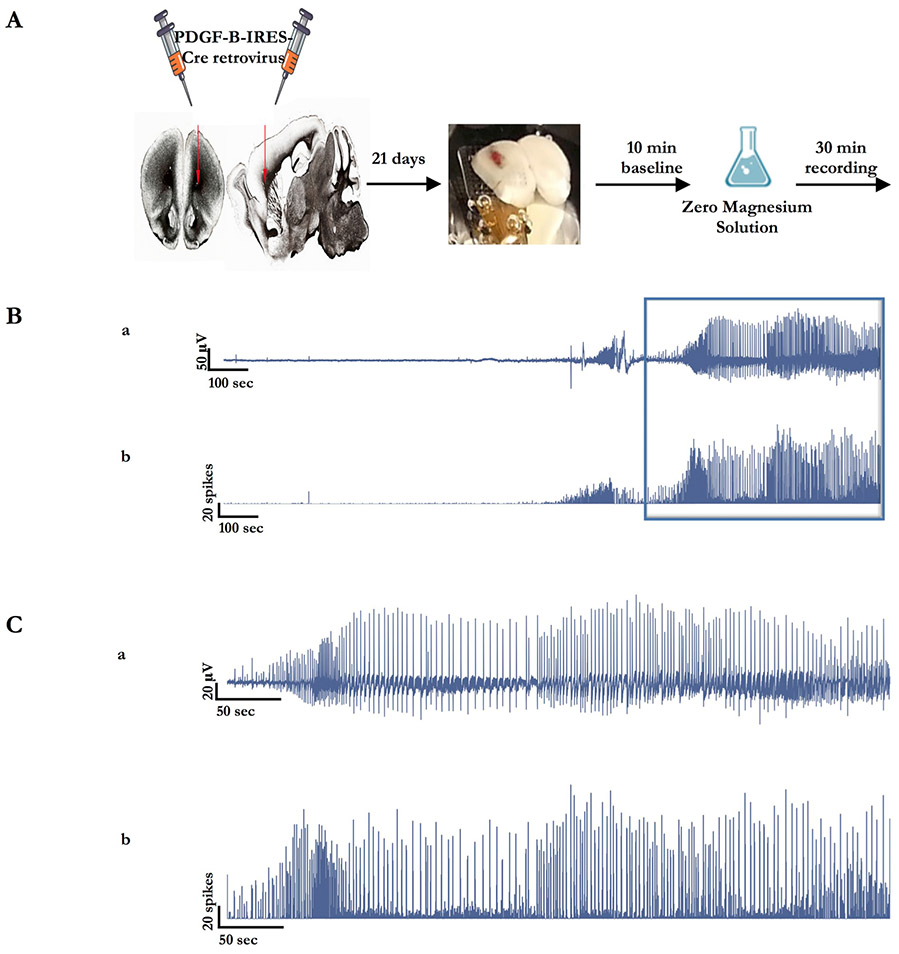
Ex-vivo model of tumor associated seizures. (A) Double floxed PTEN/p53 mice, with a YFP reporter, are injected with a PDGF-B-IRES-Cre retrovirus. 400 um thick tumor-bearing slices were harvested at 21 days and recorded on a 96 channel electrode array in artificial and zero Mg^2+^ solution. (B) LFP vs time (top, a) and binned multiunit spikes vs time (bottom, b) for a tumor-bearing slice recording. (C) Inset to (B), enlarged view of the LFP (top, a) and multiunit spikes (bottom, b) vs time for a seizure-like event.

All animal handling and experimentation was done with the approval of the Columbia University Institutional Animal Care and Use Committee. As previously described, tumor formation was induced by injecting PDGF-B-IRES-Cre retrovirus into the subcortical white matter of adult mice with PTEN^lox/lox^ p53^lox/lox^ genes. (Lei et al., 2011; Sonabend et al., 2014) We used the coordinates of 2.1mm lateral, 2.2 mm rostral, and 1.1mm deep with the bregma as the reference point (Lei et al., 2011). Mice with these retrovirus-induced tumors develop spontaneous behavioral seizures as early as 3 weeks post injection. Acute brain slices were prepared from tumor-bearing mice (*n* = 3), sacrificed at 21 days post-injection and age-matched controls (*n* = 3) (Fig. 1A). Coronal slices, 400 μm thick, were cut using a Leica VT1000S vibratome (Nussloch, Germany) while in ice-cold solution [in mM: 210 Sucrose, 10 Glucose, 2.5 KCL, 0.5 CaCl_2_, 7 MgCl_2_, 26 NaHCO_3_, 1.25 NaH_2_PO_4_]. The slices were incubated at 35 °C for 18 min and then transferred 22 °C for a minimum of 42 min prior to recordings. Contents of the incubation solution are listed as follows [in mM: 1.5 MgCl_2_, 125 NaCl, 26 NaHCO_3_, 10 dextrose, 2.5 KCL, 2 CaCl_2_, 1.25 NaH_2_PO_4_].

### 2.2. Slice recordings

Slices were placed on top of a 4 × 4 mm MEA with 96 (10 × 10 electrode grid, no electrodes in the corners) 1 mm penetrating micro-electrodes with 0.4 mm orthogonal interelectrode spacing (BlackRock Microsystems, Inc., Salt Lake City, UT) (Fig. 1A). Recordings were performed across the entirety of the slice including both cortical and subcortical regions. Recordings were performed for 10 min in artificial CSF (aCSF) [in mM: 1.5 MgCl_2_, 125 NaCl, 26 NaHCO_3_, 10 dextrose, 5 KCL, 2 CaCl_2_, 1.25 NaH_2_PO_4_], followed by 30 min in zero Mg^2+^ solution. Thus, our analysis was limited to early seizure-like events (SLEs), which better reflect clinical conditions. Signals from the MEA were acquired continuously at 30 kHz per channel (0.3 Hz –7.5 kHz bandpass), with 16-bit precision and a range of +/− 8mV.

### 2.3. Data processing and statistical analysis

The raw MEA signals were bandpass filtered into two frequency bands: multiunit activity (MUA, 500 Hz – 5 kHz, 512th order, window-based FIR filter) and local field potential (LFP, 2 - 50 Hz, 512th order, window-based FIR filter). Both filtered data streams were visually reviewed to exclude channels and time periods with excessive artifact. Multiunit spikes were detected from the MUA band using a threshold crossing method (Quiroga et al., 2004). Multiunit spikes were identified as signal peaks > 5 standard deviations (S.D.) above or below the mean for each channel. Detection refractory period was set to be 1 ms, to minimize the detection of noise overriding an action potential peak. Samples of the multiunit band were reviewed from 5 channels in each slice to confirm that action potential waveshapes correlated with detected multiunit timestamps (Supplementary Fig. S1). Channels that failed to detect more than one multiunit spike per minute were excluded from further analysis. All analyses were performed offline in the MATLAB (MathWorks) environment using custom scripts. Data that were not normally distributed were analyzed using the Wilcoxon rank sum test. Significance was set at *P* ≤ .05.

### 2.4. Identification of ex vivo seizure like events

There were two sequential criteria used to identify SLEs, of which both had to be met. Multiunit timestamps from active channels were used to construct a spike train. Whole-slice multiunit firing rates were then estimated by convolving this spike train with a 400 ms S.D. Gaussian kernel. Both in vitro(Reyes-Garcia et al., 2018) and in vivo (Liou et al., 2018) SLEs have been described as having a duration of > 5 s. Thus, periods of sustained firing > 1 standard deviation above the mean for the entire recording, for at least 5 s, were considered potential SLEs. To systematically review the LFP of potential SLEs, we modified the method employed by Bink et al (Bink et al., 2018). An electrode representative of the epileptiform activity present in the slice was used as the detector channel. The windowed standard deviation (standard deviation of the LFP signal within a sliding window 2 s in length shifting forward in steps of 1 s for the duration of the recording) was calculated. The threshold used to separate epileptiform and non-epileptiform events was set to 5 times the standard deviation of the LFP during the first two minutes of the recording. Two board-certified neurophysiologists (CAS and LMB) visually reviewed the LFP of all candidate regions for final confirmation of all SLEs.

### 2.5. Identification of recruited channels and the ictal wavefront

Channels with a minimum average firing rate of 5 multiunit spikes per second during the SLE, and with changes in their LFP similar to the detector channel, were considered to be recruited to the SLE. Spatially distinct regions—defined as regions with at least one intervening non-recruited electrode in between—activated during the same SLE were treated as separate seizure foci. All seizure foci had to contain at least 2 contiguous channels. For each of these channels, instantaneous multiunit firing rates were estimated by convolving their spike train with a 400 ms S.D. Gaussian kernel. The width of this kernel emphasized the tonic firing of the ictal wavefront as opposed to the faster bursting seen later in the SLE, thus allowing for the identification of recruitment times. The time of recruitment in each channel was determined by identifying the time of the peak-firing rate during the SLE. Recruited channels were then ordered sequentially based on the time at which the maximum peak occurred. The distance between the first recruited channel and each successive channel in this index was calculated. Wavefront speed was then determined from a linear fit of peak times relative to the first recruited channel versus the corresponding distance (Schevon et al., 2012). The Fano Factor – the variance of the multiunit activity or spiking, divided by the mean – was calculated for each slice. This is a measure of neuronal firing variability across an extended territory and has been applied to human multiunit data in the peri-ictal period to capture the diversity of neuronal firing patterns seen during focal seizures (Schevon et al., 2012; Truccolo et al., 2011).

### 2.6. Histologic analysis, antibodies, and microscopy

After the recording, acute brain slices were carefully removed from the array and fixed in 4% paraformaldehyde in phosphate buffered solution overnight (PBS) at 4 °C. 40 μm thick sections were cut using a vibratome. For immunohistochemical analysis free floating sections were incubated in goat serum(30 min, room temperature) and then with primary antibody for the pan-neuronal marker NeuN (mouse monoclonal, 1:100, MAB377, Millipore-Sigma, MA, RRID:AB_2298772) overnight at 4 °C. Secondary Alexa Fluor 594 conjugated antibody (1:300, Invitrogen, OR, RRID:AB_2534095) was applied with DAPI for 1 h at room temperature. Blocking serum, primary antibodies and secondary antibodies were applied in 0.3% Triton X-100 in PBS. Glioma cells were identified on the basis of endogenous YFP fluorescence.

Z-series confocal images were obtained using an inverted micro-scope (Eclipse Ti, Nikkon, Japan) under a 20 × air objective (N/A 0.75) (Nikkon, Japan) with 7 μm incremental step. Serial images were obtained with 10% overlap and then stitched together (NIS Elements, Nikkon, Japan) to obtain an image of the whole slice. Z-series were then stacked together to generate the max-intensity projection. All confocal images shown were projected views. These images were exported and processed in Fiji(Schindelin et al., 2012) and Matlab.

**Fig. 2.**
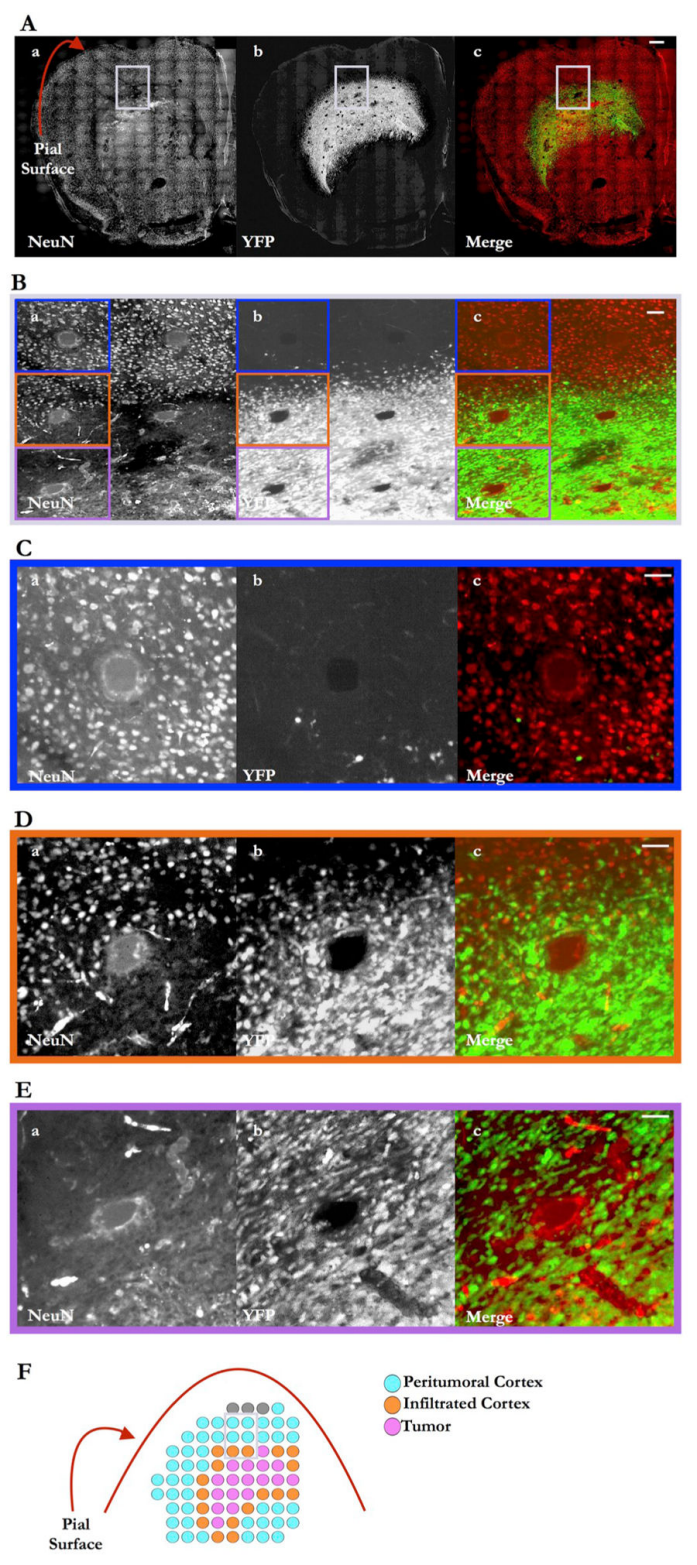
Regional variation in the histologic features of tumor-bearing slices. (A) Low power immunofluorescence micrograph of a tumor-bearing slice labeled for (a) NeuN positive neurons, (b) YFP positive neurons, and (c) merge. (B) Magnified view of three contiguous electrodes in the peritumoral cortex (blue), infiltrated cortex (red), and tumor (purple). Enlarged views demonstrating the variation in cellular composition at these electrodes in the (C) peritumoral cortex, (D) infiltrated cortex, and (E) tumor regions. (F) Cartoon representation of the tumor-bearing slice, electrodes are color-coded based on the regional histologic features, blue, red and purple represent peritumoral cortex, infiltrated cortex, and tumor, respectively. Scale bars: (A) 400 μm, (B) 100 μm, and (C-E) 50 μm.

The location of each microelectrode was identified on every slice. A 0.16mm^2^ area was defined around each electrode and the histology within this region was classified as peritumoral, infiltrated, or tumor, based on the following criteria. Areas with many NeuN + neurons and only rare YFP + glioma cells were classified as peritumoral, areas with many YFP + glioma cells intermingled with many NeuN + neurons were classified as infiltrated, and areas with many YFP + glioma cells and rare NeuN + neurons were classified as tumor (Fig. 2).

## 3. Results

### 3.1. Tumor slices display greater hyperexcitability than controls

**Fig. 3.**
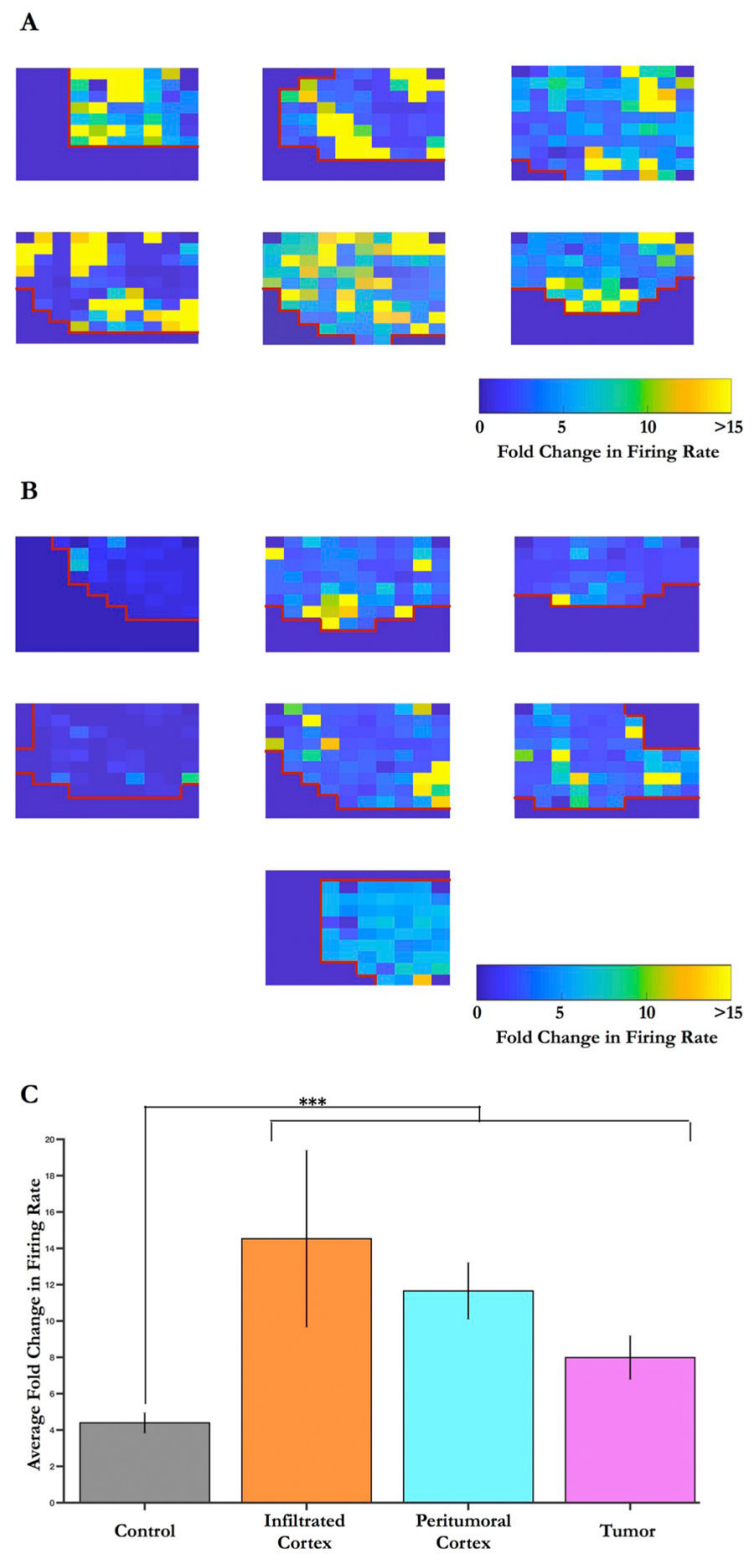
Change in firing rate following addition of zero Mg^2+^ solution. Heatmaps displaying the change in firing rate following the addition of zero Mg^2+^ solution for (A) tumor-bearing and (B) control slices. Red lines indicate the pial surface. (C) Average fold change in the firing rate in electrodes in control, peritumoral cortex, infiltrated cortex, and tumor regions. Bar data represent mean ± SEM, *** *P* < .0001. Wilcoxon rank sum test.

Electrophysiologic recordings were performed in 6 tumor slices (3 mice; mean number of active channels: 71, range: 47–93) and 7 control slices (3 mice; mean number of active channels: 63, range: 44–77). Despite the tumor-bearing mice exhibiting spontaneous behavioral seizures, there was no evidence of spontaneous SLEs during baseline aCSF recordings in either the tumor or control slices. The addition of zero Mg^2+^ solution reliably increased neuronal activity in all slices, as evidenced by increased amplitude in the 2–50 Hz frequency band and number of multiunit spikes detected from the 500 Hz – 5 kHz band (Fig. 1B and C). The slice was positioned on the array in a fashion that would allow us to capture information from channels located in areas with different histologic features. In the tumor slices we were able to consistently record from peritumoral cortex, infiltrated cortex, and tumor (Fig. 2). In total we sampled 222 peritumoral, 80 infiltrated and 97 tumor sites across the all tumor-bearing slices and 430 sites across all control slices. Increases in the firing rate following perfusion with zero Mg^2+^ solution were widespread and variable, but there was no significant difference in the change in the multiunit firing rate between the three histologic regions. However, all three types of histological regions showed significantly greater increases in firing rates when compared to the control slices (11.65, 14.52, 7.98, vs 4.39, *p*-value < .0001, Wilcoxon Rank Sum) (Fig. 3).

### 3.2. Characterization of zero Mg^2+^ induced seizure like events

**Fig. 4.**
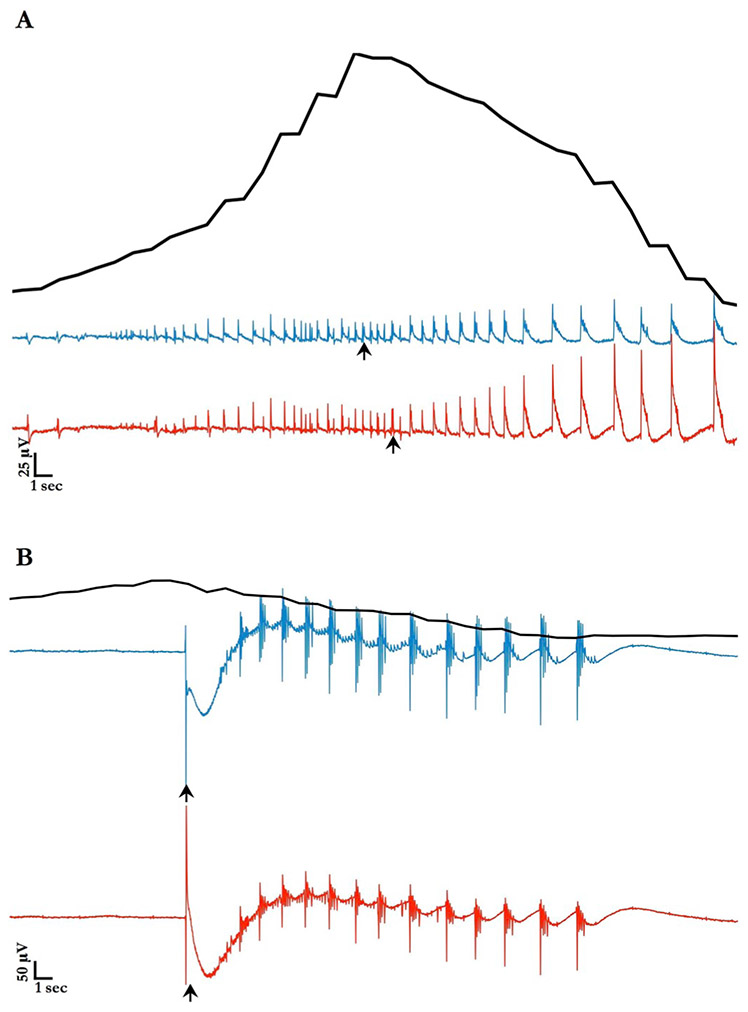
Zero Mg^2+^ seizure-like event patterns in ex-vivo slices. (A) Low voltage fast onset and (B) hypersynchronous seizure onset patterns for two contiguous channels (red and blue traces). Recruitment time for each channel denoted by the black arrow. Corresponding Fano Factor for the slice indicated by the black line, showing the increase from baseline during the SLE. [Single column].

Both the MUA and LFP were used to identify SLEs, as by definition LFP is sensitive but not specific for the presence of ictal events. We identified 40 events in the tumor slices and 5 events in the control slices (p-value = .0105, Wilcoxon Rank Sum). Based on the LFP pattern the events were segregated into two types. The first event type (4 of 45 events; 3 in tumor slices, 1 in a control slice) demonstrated the low voltage fast (LVF) onset pattern in LFP, characterized by focal beta or gamma range (> 12 Hz) oscillations at onset (Fig. 4A). The remaining 41 events were characterized by short bursts of repetitive widespread, high amplitude low frequency discharges (Fig. 4B). These resemble a type of hypersynchronous onset described in some lesional epilepsy patients (Perucca et al., 2014). In the 3 cases where both onset patterns were present, the LVF-type always preceded the hypersynchronous-type. Both the hypersynchronous-type and LVF seizure onset patterns were associated with large increases in the Fano factor, indicating a high degree of spatially heterogeneous activity.

Spatially distinct seizure foci were defined for each SLE as described in Methods. Of the 40 SLEs observed in the tumor slices, 30 contained multiple spatially distinct seizure foci. In total, 84 distinct foci were detected. The propagation speed of the SLE at each focus was calculated using the peak firing in each recruited electrode (mean 8.54 mms^−1^, range 0.06–242.03 mms^−1^). In the four SLEs with a LVF onset pattern, the average propagation speed was 0.20mms^−1^ (range: 0.06–0.32 mms^−1^), while the average propagation speed in the hypersynchronous-type onset events was 9.32mms^−1^ (range: 0.30–242.03 mms^−1^). The higher speeds of the hypersynchronous events likely reflects their timing in the late recording period, when there is breakdown of feedforward inhibition with successive events in the zero Mg^2+^ model (Trevelyan et al., 2007). The end result of this is that later SLEs often resemble discharges produced in disinhibited slices and appear to travel through the tissue in an unabated fashion.

**Fig. 5.**
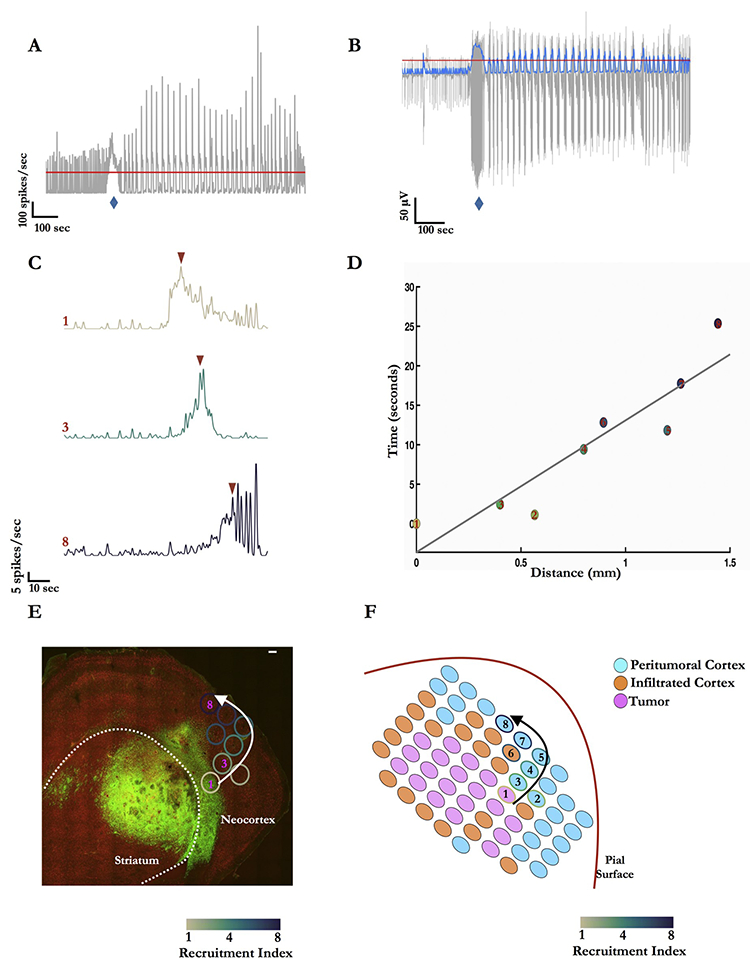
Propagation of seizure-like events in a mouse tumor-bearing slice. (A) Whole slice multiunit firing rate vs time for a tumor-bearing slice, mean firing rate indicated by red line (B) LFP for detector channel from this slice. Blue diamond indicates the SLE of interest, red and blue lines indicate the threshold and windowed standard deviation respectively. (C) Multiunit firing rates for 3 channels in a seizure focus recruited to this event, arrowheads delineate the pre-recruitment from the post-recruitment periods. (D) Scatter plot of time to recruitment vs distance from the first recruited channel. The inverse of the slope of the line of best fit indicates the propagation speed of 0.06 mms^−1^. (E) Delay map of recruitment activity for the seizure like event overlaid on a low power immunofluorescence micrograph and (F) cartoon representation of the slice. Arrows in (E) and (F) show the direction of propagation, with onset in the tumoral neocortex and spread to the surrounding peritumoral cortex. Numbers, and colorbar in (E) and (F) indicate the recruitment index for the channels. Scale bar: (E) 100 μm. Dashed white line in (E) delineates the neocortical from striatal regions.

Seizure foci comprised areas from all three histologic groups in tumor slices (Fig. 5). The onset electrodes in 53/84(63.1%) of these foci lay in the neocortex, while the remaining 31 (36.9%) localized to the striatum. In 42 of the 84 foci, the onset electrode was at the infiltrated margins of the tumor, with 31 foci lying peritumoral. Furthermore these peritumoral instances laid an average of 0.45 mm from the nearest electrode in the tumor or infiltrated margins (range 0.4–1.2 mm). This was significantly different than the average distance of the 222 actively recorded peritumoral electrodes from the nearest electrode in tumor or infiltrated margins (mean 0.72 mm, range 0.4–1.2 mm *p*-value < .000001, Wilcoxon Rank Sum). All observed SLE onset electrodes that lay within tumor regions were no > 0.4 mm away from the nearest electrode in the infiltrated or peritumoral regions.

### 3.3. Local field potential responses during seizure like events

**Fig. 6.**
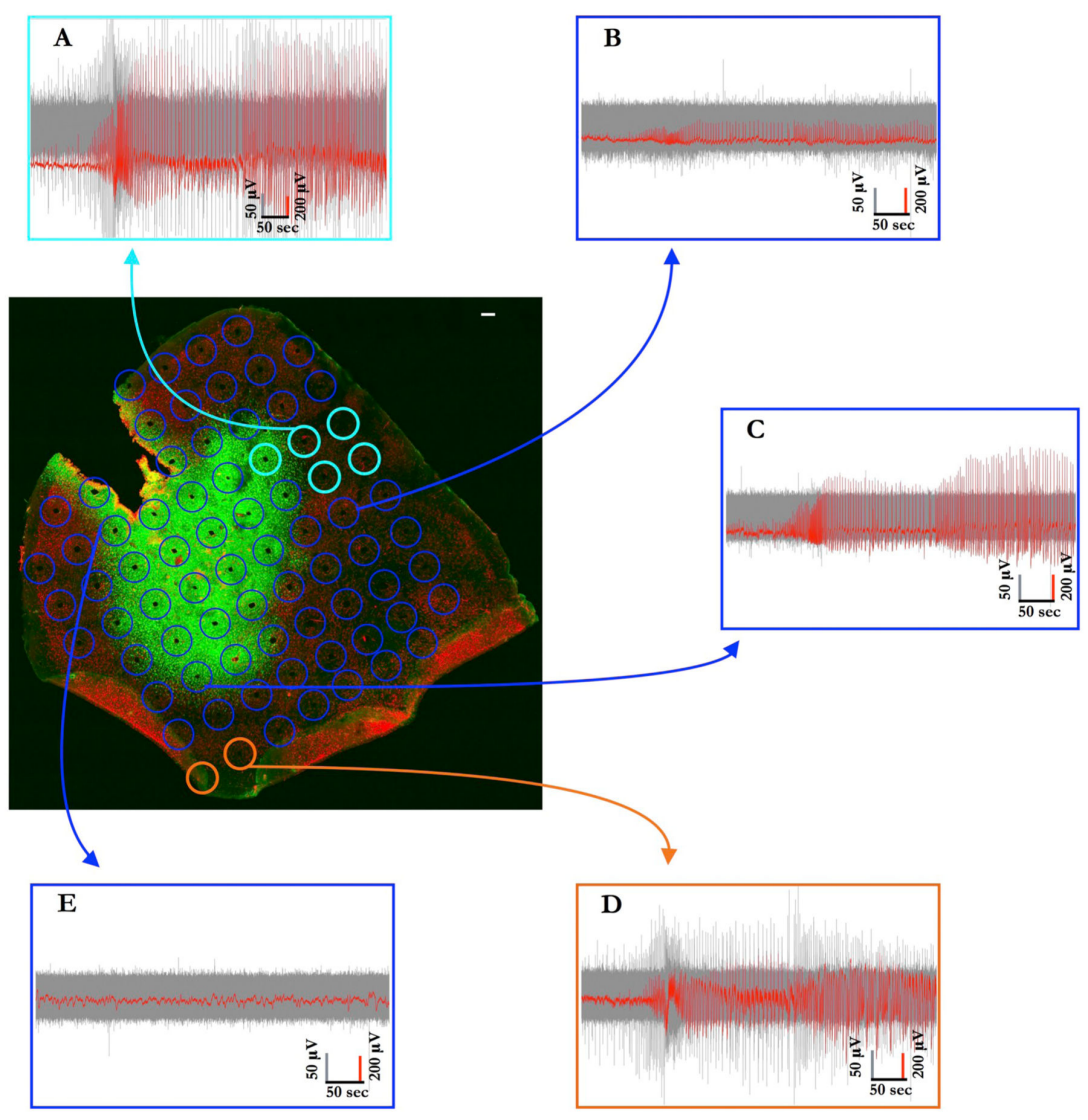
Influence of ictal activity on surrounding sites during a seizure-like event. Tumor-bearing slice with two seizure foci of recruited channels indicated by the light blue and orange channels. Low frequency (red trace) and multiunit signals (gray trace) in channels (A) and (D) display evidence of recruitment to a SLE. Low frequency and multiunit signal from non-recruited channels at various distances are displayed in (B), (C), and (E). Note that while the channels in (B) and (C) have similar low frequency signals to the recruited channels, their multiunit traces display no evidence of seizure invasion. Channel (E) demonstrates no changes in either the low frequency or multiunit bands during the event. Scale bar: 100 μm.

Previous studies of human seizure propagation in-vivo have categorized cortical regions based on whether or not they have been actively recruited into ictal events (Schevon et al., 2012). Core territories display multiunit bursts temporally locked to the LFP discharges. In contrast, penumbral territories are regions characterized by large amplitude LFP changes that are not phase-locked to concomitant low level neuronal firing. The relatively low number of units detected in ex vivo slices prohibits robust multiunit spike-LFP phase correlation analysis. Despite this, the presence of core and penumbral regions was evident based on inspection of both the LFP signature and the multiunit bands. While large amplitude LFP changes were present in both core and penumbra sites, putative core regions also demonstrated simultaneous increases in the multiunit band (Fig. 6). This shows that these SLEs may produce broad changes in the field potentials throughout the tissue, without actual ictal invasion, and underscores the importance of using the multiunit band to identify seizure onset zones.

## 4. Discussion

Methods to simultaneously investigate the regional electrophysiologic and histologic complexities of lesional epilepsy are essential. TAE is of particular interest as there is increasing evidence to suggest that there may be a reciprocal relationship between seizures and glioma growth (Venkatesh et al., 2015). Previous studies have delineated many of the mechanisms underlying the inherent epileptogenicity of gliomas (Buckingham et al., 2011; Campbell et al., 2015; Pallud et al., 2014; Robert et al., 2015; Tewari et al., 2018; Ye and Sontheimer, 1999). Building upon these efforts, we present a frame-work for how the spatiotemporal relationship of ictogenesis and ictal propagation can be investigated with respect to the varying cellular composition and histologic landscape seen in glioma. This methodology can also potentially be applied to other types of lesional epilepsy, including tuberous sclerosis, focal cortical dysplasia, various types of epilepsy inducing low grade gliomas and mesial temporal sclerosis.

In acute brain slices, SLEs rarely occur spontaneously, and most experimental methods use electrical stimulation or pharmacologic agents to induce and study epileptiform activity. Zero Mg^2+^ solution is a well-established chemoconvulsant that works by enhancing excitation as the extracellular Mg^2+^ that normally blocks the NMDA channels is removed. Importantly, unlike other agents such as picrotoxin or bicuculine, this method preserves the local inhibitory network, thus allowing for meaningful assessment of ictal recruitment patterns that are modulated by feedforward inhibition. Previous studies have shown that prolonged exposure to zero Mg^2+^solution produces continuous rhythmic discharges lasting less than one second in duration as opposed to propagating ictal events, giving cause to limit our experiments to 30 min (Anderson et al., 1986; Dulla et al., 2018). The major disadvantage of using this chemoconvulsant, however, is that it there is widespread increase in the level of excitatory glutamatergic drive across the slice. This precludes a clear picture of the spatial arrangement of excitatory effects across the different histologic territories. Future work could potentially address this by assessing the response to focal cortical stimulation at electrodes in each territory.

Seizures are typically defined clinically by the presence of a stereotypic semiology and concomitant changes on EEG. Comparatively, there is an inherent difficulty in determining whether a given electrophysiological event in a slice should be described as a seizure due to the absence of a clinical or behavioral correlate, and therefore definitions are predicated on electrographic changes alone. To that end, we chose to consider both the MUA and low frequency bands when defining potential ictal events in this model. We combined this approach with the modified method of automated event detection used by Bink et al. (2018). This technique takes advantage of the increased amplitude in the low frequency bands to identify putative ictal events. This is comparable to the standard EEG used to evaluate clinical seizures in human epilepsy. Such a combined approach is necessary for accurate correlations with the histologic landscape, as recorded field potentials do not necessarily reflect the process of spatial evolution of ictal events. Additionally propagating ictal events were found in some instances to involve more than a single noncontiguous focus, further complicating assessments of large-scale ictal propagation. Multiple foci are commonly observed in clinical epilepsy, but are not commonly observed in acute seizure models. Our use of a chronic model, together with assessment of both LFP and MUA to define seizure territories, revealed that multiple activating sites may be common in the chronic epileptic condition.

In-vivo studies of human patients undergoing intracranial electroencephalography for non-neoplastic lesional epilepsy have revealed a variety of seizure onset patterns of which LVF-type and hypersynchronous-type are the two most common (Perucca et al., 2014). LVF onset seizures occurred across a variety of pathologies, while hypersynchronous seizures were only seen in cases of mesial temporal sclerosis (MTS) (Perucca et al., 2014). In our ex vivo experiments on neoplastic tissue we observed both types, with the majority of SLEs being the hypersynchronous-type. Computational models of these two seizure types have shown that LVF onset is characterized by localized activity that slowly invades the tissue over time. In contrast, the hypersynchronous events reflect broad increases in the excitability of the surrounding tissue allowing it to sustain ictal events (Wang et al., 2017). Bearing this in mind, the predominance of the hypersynchronous events in this model could certainly be secondary to the chemoconvulsant utilized, given the widespread effects that zero Mg^2+^ solution has on excitatory tone throughout the slice. However, the findings that these SLEs are significantly more frequent in the tumor-bearing slices, and predominantly arise at the infiltrated margins of the tumor, indicate that the glioma's infiltrated brain tissue is hyperexcitable compared to control brain slices. While beyond the scope of this study, it is likely that this result reflects a multitude of glioma-induced changes in the surrounding infiltrated and peritumoral cortex, which may affect regional inhibitory and excitatory function and produce focal regions of increased seizure susceptibility. Other groups have described these changes at length, and they include reduced levels of parvalbumin interneurons(Tewari et al., 2018), decreased expression of KCC2(Pallud et al., 2014), tumor-mediated glutamate release (Buckingham et al., 2011; Ye and Sontheimer, 1999), and reduced glutamate uptake(Yuen et al., 2012). These changes have been shown to occur at the infiltrated margin of glioma and may explain why the onset electrodes seen in our model localize to the periphery of the lesion in each slice. However, our method clearly demonstrates that seizure susceptibility is not uniform even within tumor sub-regions, indicating that tissue studies predicated on sites differentiated by electrophysiological characteristics may provide more detailed insight into the process of ictogenesis.

Unlike patch clamp techniques, MEAs have made it possible to perform extracellular recordings from a large number of neurons simultaneously. Previous studies have taken advantage of this to understand the spatiotemporal dynamics of epilepsy (Schevon et al., 2012; Smith et al., 2016; Truccolo et al., 2011). Voltage sensitive dyes have been used to investigate spreading epileptiform activity in ex vivo models of glioma (Buckingham et al., 2011; Robert et al., 2015). This technique reflects the changes in the LFP (Wu et al., 1999) but unlike the MEA has a poorer temporal resolution and is unable to provide concomitant access to the multiunit neuronal behavior which characterizes seizures. Using MEAs allowed us to show that the presence of a tumor produces multiple spatially distinct seizure foci. This effectively recapitulates a known facet of lesional epilepsy and illustrates why subtotal resection often does not lead to seizure freedom.

## 5. Conclusions

In summary we have described an ex vivo MEA based approach to evaluate both the histological and electrophysiologic characteristics of areas recruited to SLEs. To the best of our knowledge this is the first study of its kind to address the spatiotemporal dynamics of seizures in this form of chronic lesional epilepsy. This strategy revealed that tumor-bearing slices have greater seizure proclivity than controls, contain multiple noncontiguous seizure foci, and have SLEs arising from the infiltrated margins of the tumor. Future work could harness the MEAs potential to isolate single units(Merricks et al., 2015) to ascertain the behavior of different neuronal populations during ictogenesis in the peritumoral environment. Another could be to test the efficacy of anti-epileptic and other disease-modifying drugs in this setting. And finally, the technique is flexible enough to be combined with post-hoc histologic analysis, genetically encoded indicators of neuronal activity, and high-throughput sequencing, providing even greater insight into the molecular basis of lesional epilepsy.

## Supplementary Material

Supplementary Figure 1

## Data Availability

The data sets and code used to interpret them are available from the corresponding author on reasonable request.
