# Supplementary figures and images for: Ex vivo multi-electrode analysis reveals spatiotemporal dynamics of ictal behavior at the infiltrated margin of glioma
Source: Neurobiol Dis. Author manuscript; Available in PMC 2021 May 25. (PMC8147009; doi:10.1016/j.nbd.2019.104676)

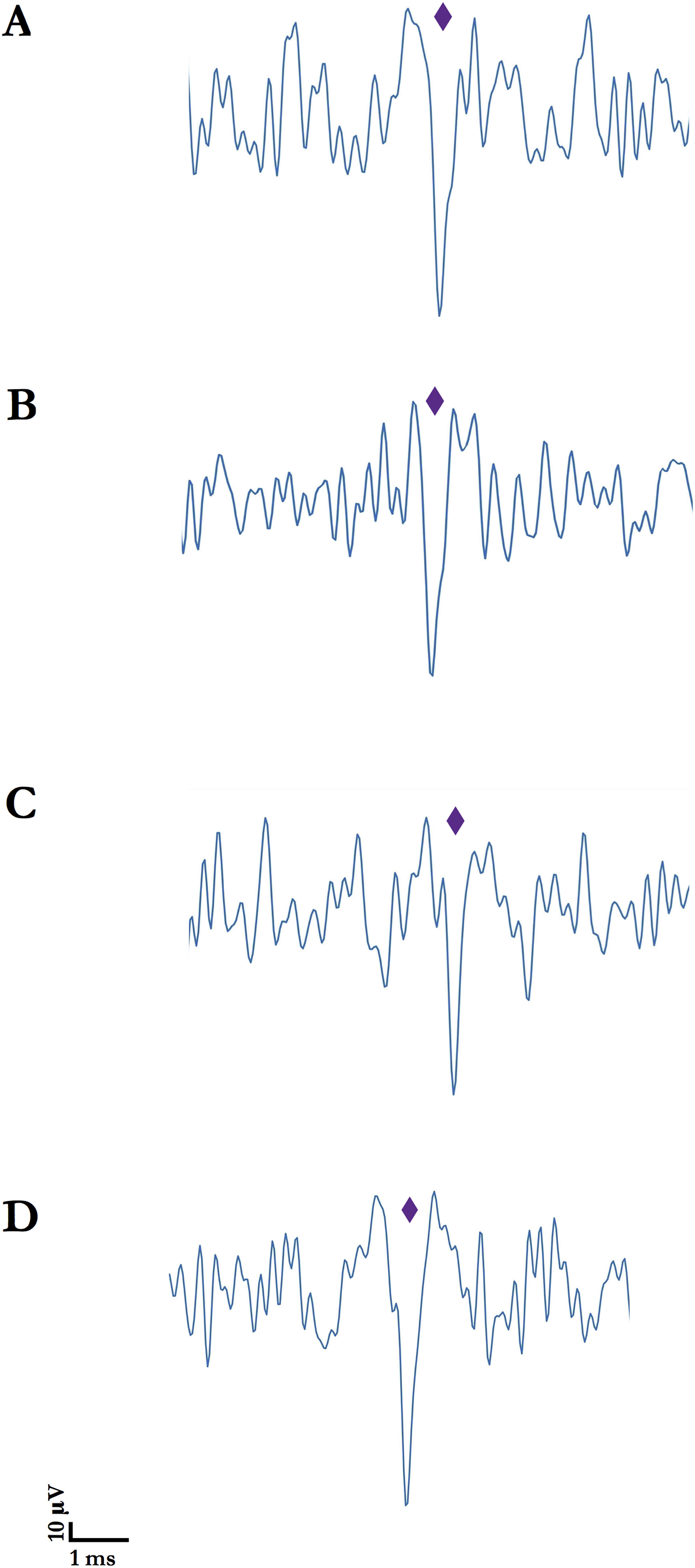

Supplement: Supplementary Figure 1 [file NIHMS1697777-supplement-Supplementary_Figure_1.jpg]
